# 775. Needs Assessment to Support Continuing Education and Training Modalities for High-Consequence Pathogen Preparedness in Pediatric and Obstetric Populations

**DOI:** 10.1093/ofid/ofad500.836

**Published:** 2023-11-27

**Authors:** Clayton Mowrer, Laura J Fischer, John Horton, Nichole L Huff, Chimora Imonugo, Andi L Shane, Lisa Stone, Kari Simonsen

**Affiliations:** University of Nebraska Medical Center/Children's Hospital and Medical Center, Nebraska; University of Nebraska Medical Center, Omaha, Nebraska; Emory Healthcare, Atlanta, Georgia; NETEC, Jersey City, New Jersey; Emory University School of Medicine, Atlanta, Georgia; Emory University School of Medicine and Children's Healthcare of Atlanta, Atlanta, GA; National Emerging Special Pathogens Training and Education Center, Atlanta, Georgia; University of Nebraska Medical Center, Omaha, Nebraska

## Abstract

**Background:**

High-consequence pathogens present complex challenges in United States (US) healthcare, with knowledge gaps existing in the provision of safe and effective treatment. The National Emerging Special Pathogens Training and Education Center (NETEC) aims to standardize guidance and care coordination across the healthcare continuum for suspected or confirmed infection with high-consequence pathogens through education, training, research, monitoring, evaluation, and funding. The NETEC Special Populations Committee applies this mission to pediatric and obstetric care.

We conducted a needs assessment to optimize education and training opportunities for healthcare teams related to high-consequence pathogen preparedness for children and pregnant women.

**Methods:**

Stakeholders received a needs assessment survey via email, social media, and NETEC website from 10/3/22-11/15/22. Questions targeted demographics, topics of interest for continuing education within the special populations: pathogen awareness, frontline care, infection prevention, and emergency preparedness, and learning modality preferences.

**Results:**

Of the 20,600 impressions, the survey was completed by 145 persons (with 97 additional partial completions). The use of targeted emails had the highest engagement, with a 25% open rate (29% industry average) and click-through rate of 18% (5% industry average). Respondents were located across the US, mainly in academic healthcare settings (46%). Infection Preventionists (30%) represented the greatest proportion, but many institutional roles were reported (Table 1). Responses to educational topics were widely distributed (Table 2). Webinars (77%), in-person training (64%), and brief written summaries or updates (54%) were the most common preferred learning modalities (Figure 1).

**Figure:**
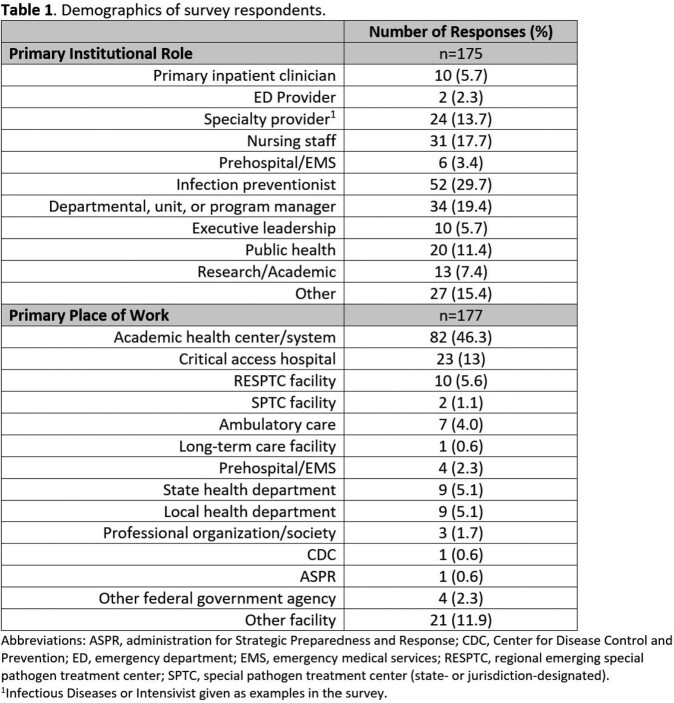

**Figure:**
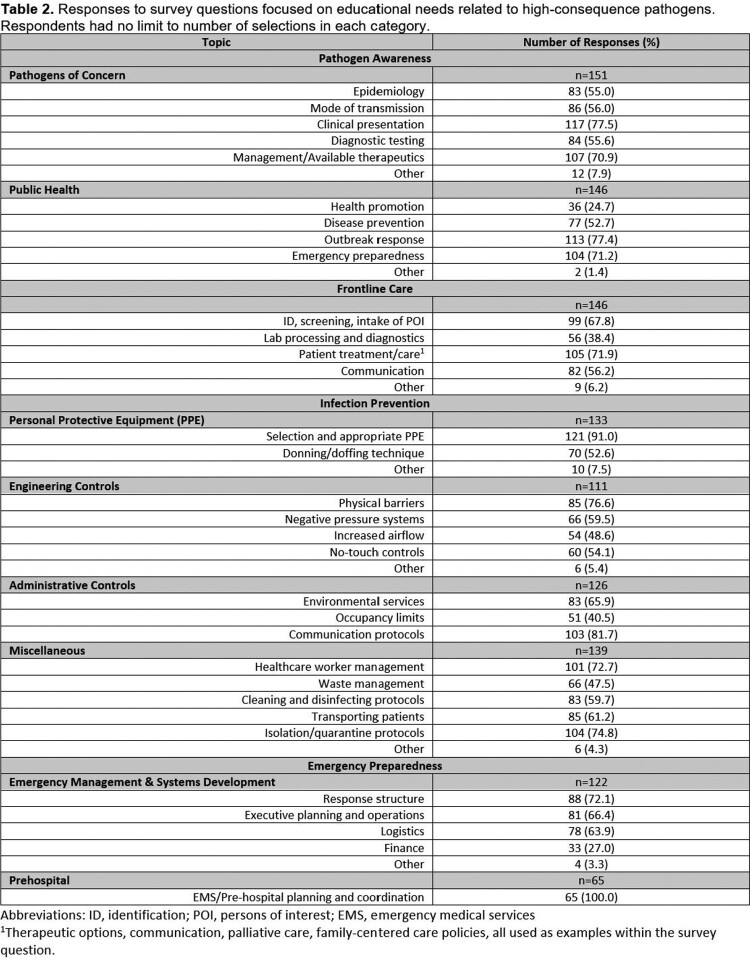

**Figure:**
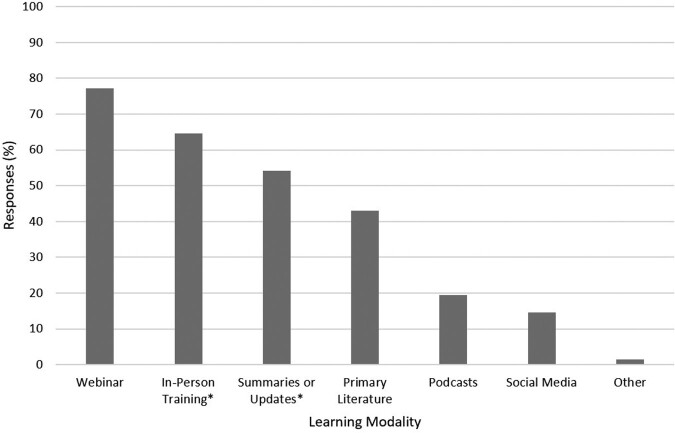

**Conclusion:**

Myriad healthcare team members are involved in the care of pediatric and obstetric patients with high-consequence pathogens. They represent an engaged group of NETEC stakeholders that, despite recent experience with the SARS-CoV-2 pandemic, express interest in continuing education on topics related to high-consequence pathogens across the care continuum via interactive, engaging, and collaborative learning modalities, led by subject matter experts.

**Disclosures:**

**Andi L. Shane, MD, MPH, MSc**, International Scientific Association for Probiotics and Prebiotics: Travel and lodging support to attend an international meeting June 2022|Pediatric Infectious Disease Society (PIDS): Stipend for serving as Deputy Editor **Kari Simonsen, MD, MBA**, Astra-Zeneca: Grant/Research Support|Melinta Therapeutics: Grant/Research Support|Pfizer: Grant/Research Support

